# The associations between skin advanced glycation end-products and Framingham cardiovascular risk in different age groups

**DOI:** 10.3389/fcvm.2025.1491643

**Published:** 2025-04-08

**Authors:** Yina Wang, Shangyan Liang, Ying Zhou, Xiumei Tang, Na Ye, Weilan Huang, Xixiang Tang, Boxiong Jiang, Yunfeng Pan

**Affiliations:** ^1^ Department of VIP Medical Service Center, Third Affiliated Hospital of Sun Yat-sen University, Guangzhou, Guangdong, China; ^2^ Department of Health Management Center, Third Affiliated Hospital of Sun Yat-sen University, Guangzhou, Guangdong, China; ^3^ Department of Rheumatology and Immunology, Third Affiliated Hospital of Sun Yat-sen University, Guangzhou, Guangdong, China

**Keywords:** skin advanced glycation end-products, Framingham cardiovascular risk score, atherosclerotic cardiovascular disease, different age groups, skin autofluorescence

## Abstract

**Objective:**

Advanced glycation end-products (AGEs) may contribute to the pathogenesis of atherosclerotic cardiovascular disease (ASCVD), potentially influencing its development and progression differently at various life stages. This study aimed to elucidate the associations between AGEs and the risk of ASCVD across different age groups.

**Methods:**

In this cross-sectional study, 1,240 subjects were enrolled and divided into three groups (Group Ⅰ, 20–39 years old, *n* = 468; Group Ⅱ, 40–59 years old, *n* = 471; Group Ⅲ, 60–79 years old, *n* = 301). Skin AGEs were measured by skin autofluorescence (SAF). ASCVD risk was assessed by a validated Framingham risk score calculator. Other proven ASCVD risk factors were also measured, including glycosylated hemoglobin, uric acid, lipid profile, homocysteine, and cystatin C.

**Results:**

An increasing trend in skin AGEs was observed from Group Ⅰ to Group Ⅲ. Skin AGEs were significantly associated with ASCVD risk in all subjects (OR 1.029, 95% CI 1.003–1.056, *P* = 0.018), independent of some of the proven cardiovascular risk factors. This association was particularly significant in individuals aged 40–59 and 60–79 (OR = 1.047, 95% CI: 1.025–1.069; OR = 1.022, 95% CI: 1.002–1.042; both *P* < 0.05). ROC analysis showed that skin AGEs predicted the diagnosis of medium or high ASCVD risk in the pooled group, Group Ⅱ, and Group Ⅲ.

**Conclusion:**

Our study substantiates that skin AGEs play an important role as an independent risk factor for ASCVD, highlighting their significance beyond traditional risk assessment models, particularly in middle-aged and older populations.

## 1 Introduction

Atherosclerotic cardiovascular diseases (ASCVD) remain the leading cause of death globally, posing a significant public health challenge (1). The development of ASCVD is well acknowledged as multifactorial, involving a wide range of factors from lifestyle to genetics. Among these factors, advanced glycation end-products (AGEs) have garnered increasing attention in recent years. AGEs are complex molecules formed through the non-enzymatic reaction of reducing sugars with proteins or lipids, accumulating over time in human tissues, especially in the skin (2).

Studies have indicated that the formation and accumulation of AGEs are closely associated with the progression of various chronic diseases, particularly diabetes, renal diseases, neurodegenerative diseases, and cardiovascular diseases (3, 4). In the cardiovascular domain, AGEs contribute to increased cardiovascular risk by promoting inflammatory responses, enhancing oxidative stress, damaging endothelial function, and accelerating vascular stiffening, thereby increasing the risk of cardiovascular events (5). Moreover, AGEs, through their interaction with the receptor for AGEs (RAGE), activate a series of signaling pathways, further promoting the development of cardiovascular diseases (6).

Emerging evidence highlights that AGE accumulation follows a non-linear trajectory across the human lifespan, modulated by both intrinsic aging processes and extrinsic environmental exposures (7). While basal levels of AGEs exist in healthy youth due to physiological metabolism, their deposition accelerates markedly after the fourth decade of life (7). Three interconnected mechanisms drive this age-dependent escalation, namely, declining detoxification capacity (8), cumulative metabolic insults (9), and senescence-associated secretory phenotype (10). Given these findings, ASCVD risk may exhibit age-stratified patterns, necessitating further clinical investigation to delineate these dynamics across different life stages.

Although traditional cardiovascular risk scoring models, such as the Framingham cardiovascular risk score, have been extensively used in clinical practice to assess an individual’s 10-year ASCVD risk, these models primarily focus on traditional risk factors such as age, gender, systolic blood pressure (SBP), total cholesterol, high cholesterol, smoking, hypertension, and diabetes (11). These traditional scoring systems might not fully predict the risk for all individuals, especially in those where traditional risk factors are not prominent, but the levels of AGEs are elevated.

With the advancement of non-invasive biomarker measurement technologies, the measurement of skin AGEs has emerged as a research focus, as it provides a potential method to assess the accumulation of AGEs in the body and the associated cardiovascular risk (12). Integrating the measurement of skin AGEs into existing cardiovascular risk assessment models might help to improve the accuracy of risk predictions. The accumulation of AGEs is a gradual process, potentially influencing the pathogenesis of ASCVD differently at various life stages. Therefore, exploring the relationship between skin AGEs and the Framingham CVD risk score, particularly with an age-stratified approach, not only can enhance our understanding of the risk factors for ASCVD but can also help improve existing risk assessment models to be more personalized and predictive, thereby promoting more effective prevention and treatment strategies.

## 2 Methods

### 2.1 Study population

**Figure 1:**
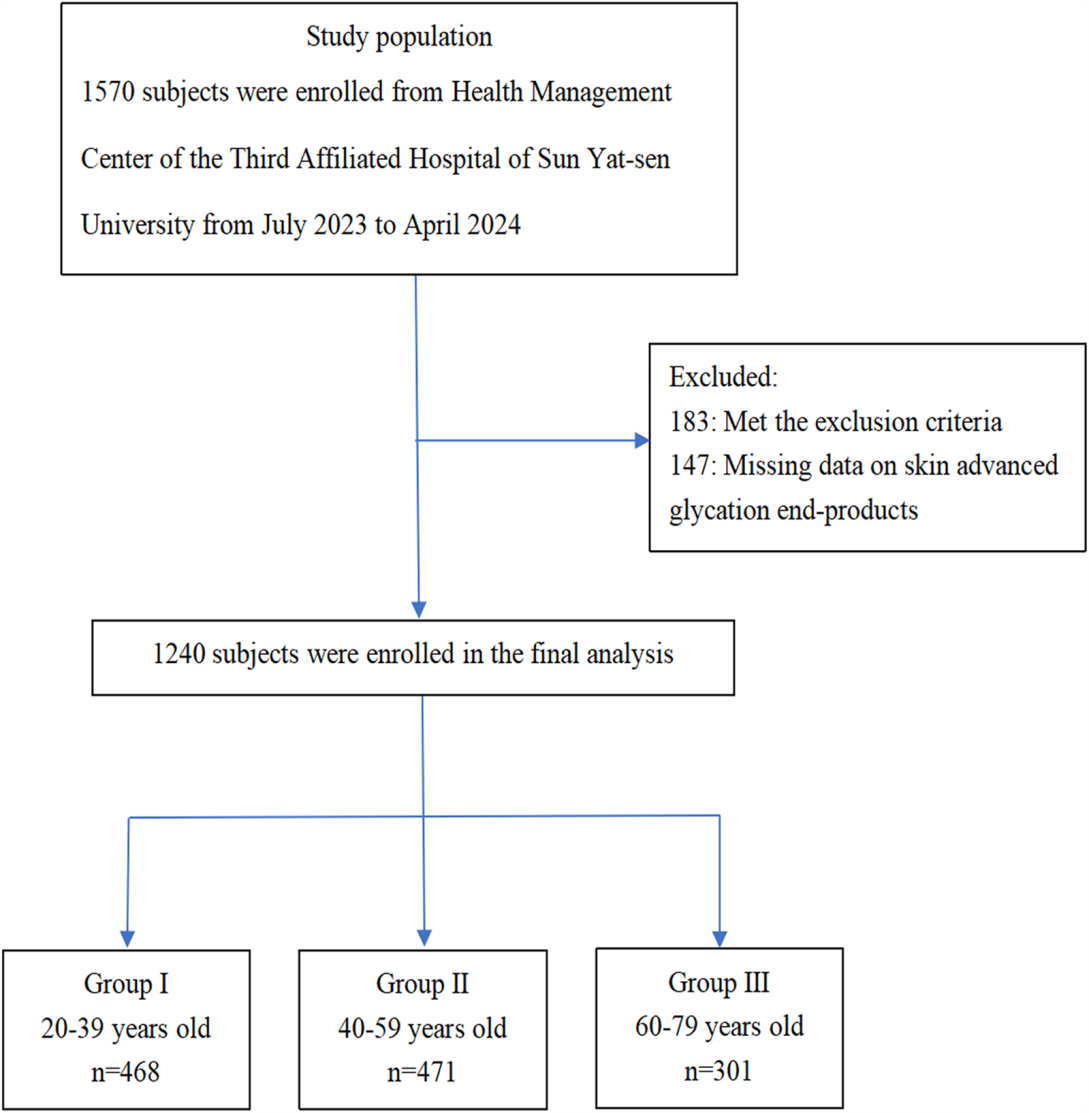
Flowchart of population enrollment.

In this cross-sectional study, 1,570 subjects were enrolled from the Health Management Center of the Third Affiliated Hospital of Sun Yat-sen University from July 2023 to April 2024. Detailed enrollment information is shown in Figure 1. All the subjects were divided into three groups with a 20-year interval to balance the age distribution: 20–39 years old, 40–59 years old, and 60–79 years old, respectively (13, 14). Subjects who met the following criteria were excluded from the study: (1) acute stress conditions such as surgery, trauma, and infection; (2) administration of hormones or other drugs that raise blood glucose levels; (3) cardiovascular diseases such as coronary heart disease, cerebrovascular disease, peripheral vascular disease, and heart failure; (4) anemia; (5) cancer; (6) severe liver insufficiency and kidney insufficiency; (7) inability to move the limbs normally because of limb, joint, or spinal diseases; and (8) pregnant or lactating women.

### 2.2 Data collection

Demographic and clinical characteristics, including gender, age, height, and weight, were obtained for all patients by a well-trained researcher. Body mass index (BMI) was calculated as body weight (kg) divided by the square of the height (m). The blood pressure levels were calculated using three consecutive blood pressure values 10 min apart taken in a sitting position. Medical history and lifestyle behavior were obtained using a unified questionnaire, including hypertension, diabetes, family history of cardiovascular diseases, medications (such as antihypertensive, lipid-lowering, and antidiabetic agents), smoking status, and alcohol consumption.

### 2.3 Laboratory testing

The following blood sample was obtained in the morning after a 10 h overnight fast. Serum concentrations of total cholesterol (TC), triglyceride (TG), low-density lipoprotein cholesterol (LDL-C), high-density lipoprotein cholesterol (HDL-C), uric acid, and plasma glucose were detected by automated enzymatic method. The concentration of serum cystatin C (CysC) was measured via particle-enhanced turbidimetric immunoassay. The concentrations of apolipoprotein A-1 (Apo-A) and apolipoprotein B100 (Apo-B) were measured by transmission turbidimetric immunoassay. The concentration of lipoprotein (a) [Lp(a)] was measured via the latex immunoturbidimetry method. All the above indexes were detected with an autonomic analyzer (Hitachi, 7600-020). Plasma homocysteine (Hcy) was measured by high-performance liquid chromatography with fluorescence (Siemens, ADVIA Centaur CP). Glycated hemoglobin A1c (HbA1c) was measured by high-performance liquid chromatography (Bio-Rad, D-100).

### 2.4 Measurement of AGEs

Skin AGEs were measured by skin autofluorescence (SAF) through a spectroscopy device (AGE Pro, Hefei Institutes of Physical Science, Chinese Academy of Sciences) (15). AGEs have fluorescent properties. The device utilized excitation light at a peak wavelength of 370 nm to stimulate AGEs in the skin, resulting in fluorescence emissions within the range of 420–600 nm. Skin diffuse reflectance in the wavelength range of 350–600 nm can also be captured to account for tissue absorption and scattering effects. The accumulation of AGEs was quantified using both the fluorescence and diffuse reflectance data processed through a proprietary algorithm. An excitation light source with a peak wavelength of 370 nm was used to illuminate a 1–4 cm section of the forearm. The subjects took the seat and put their left arm elbow joint in the fixed position of the measuring instrument groove, at room temperature in a semi-dark environment. The contact probe was placed at a normal skin site of the volar side of the left forearm, without prominent skin lesions such as blood vessels, scars, lichenification, birthmarks, and tattoos. AGE accumulation (any unit, AU) was determined as the value of skin AGEs. Three examinations were conducted by trained operators, and an average score was obtained for statistical analysis.

### 2.5 Assessment of ASCVD risk

ASCVD risk in the study was assessed by a previously validated Framingham cardiovascular risk score calculator suitable for individuals with no prior cardiovascular disease (11). The risk calculator is based on a Cox regression model with proportional risk, which incorporates age, sex, TC, HDL-C, systolic blood pressure, blood pressure-lowering medication use, diabetes status, and smoking status. It is used to estimate 10-year and lifetime risks for ASCVD of an individual, such as angina, myocardial infarction, coronary death, ischemic stroke, hemorrhagic stroke, transient ischemic attack, peripheral artery disease, and heart failure. ASCVD risk was categorized into three categories: low (<10%), medium (10%–20%), and high (>20%) risk (11).

### 2.6 Statistical analysis

Subjects were divided into three groups every 20 years old. Continuous data were presented as either mean ± standard deviation if normally distributed or median (interquartile range) if non-normally distributed. The differences in all variables across age groups were compared by one-way ANOVA, followed by the least significant difference test (LSD-t) for pairwise comparison if they were consistent with normal distribution or analyzed by Kruskal–Wallis test with Bonferroni’s method for further pairwise comparison if they were not consistent with normal distribution. Categorical data were expressed as numbers and percentages, and the differences between groups were compared by the chi-square test. Spearman's rank correlation analysis was used to assess the relationships between different continuous data. Univariate and multivariate ordinal logistic regression analyses were performed with AGEs and other cardiovascular risk factors as the independent variables and Framingham ASCVD risk categories as the dependent variables, which are expressed as odds ratios (OR) with 95% confidence intervals (CI). Notably, age, sex, TC, HDL-C, SBP, use of antihypertensive agents, diabetes status, and smoking status were excluded from the multivariate analysis because they are integral to the Framingham ASCVD risk score formula. Additionally, interaction tests were performed using ANOVA, and multicollinearity was evaluated using the variance inflation factor (VIF). To investigate potential non-linear associations between skin AGEs and ASCVD risk, the restricted cubic spline (RCS) method was employed. A receiver-operating characteristic (ROC) curve analysis was designed to identify the cutoff values (thresholds) of AGEs that best predicted ASCVD risk categories. The differences in the area under the curve (AUC) among AGEs and other variables were compared in the ROC curves using MedCalc 22.0. All other statistical analyses were conducted with SPSS 26.0 (IBM, Armonk, NY, USA) and R software (version 4.4.0). Significance was defined as *P* < 0.05.

## 3 Results

### 3.1 Clinical and biochemical characteristics

A total of 1,240 subjects were enrolled in the final analysis and divided into three groups: Group Ⅰ, 20–39 years old (*n* = 468); Group Ⅱ, 40–59 years old (*n* = 471); and Group Ⅲ, 60–79 years old (*n* = 301), respectively. As shown in Table 1, the proportions of women and SBP increased from Group Ⅰ to Group Ⅲ. There were no significant differences in diastolic blood pressure, BMI, or the use of antihypertensive, lipid-lowering, and antidiabetic agents among the three groups. The presence of diabetes and hypertension was higher in Group Ⅲ than that in Groups Ⅰ and Ⅱ, while the rate of smoking and drinking was higher in Groups Ⅰ and Ⅱ than that in Group Ⅲ. As the age ascended, HbA1c, fasting blood glucose, and Cys C gradually increased, while the estimated glomerular filtration rate (eGFR) gradually decreased. Uric acid, Apo-A, and Apo-B were higher in Group Ⅲ than those in Groups Ⅰ and Ⅱ. Hcy and Lp(a) were higher in Group Ⅲ than those in Group Ⅰ. No significant differences were observed in TG, HDL-C, and LDL-C across age groups.

**Table 1:** Clinical and laboratory characteristics.

Variables	All subjects (*n* = 1,240)	20–39 age years old (*n* = 468)	40–59 age years old (*n* = 471)	60–79 age years old (*n* = 301)	*P* for trend
Clinical characteristics					
Age (years)	47.15 ± 12.54	34.75 ± 4.03	48.17 ± 5.62***	64.85 ± 4.31***^,^^†††^	<0.001
Sex [F/M (%F)]	447/793 (36.05)	104/364 (22.22)	192/279 (40.76)*	151/150 (50.17)**^,^^†^	<0.001
SBP (mmHg)	125.45 ± 16.67	121.35 ± 13.57	123.64 ± 15.85*	134.65 ± 18.73***^,^^†††^	<0.001
DBP (mmHg)	79.43 ± 10.06	78.69 ± 9.35	79.73 ± 10.42	80.12 ± 10.52	0.054
BMI (kg/m^2^)	24.45 ± 3.42	24.43 ± 3.67	24.52 ± 3.41	24.38 ± 3.05	0.817
Smoking [*n* (%)]	153 (12.34)	77 (16.45)	53 (11.25)	23 (7.64)*^,^^†^	<0.001
Alcohol drink [*n* (%)]	172 (13.87)	69 (14.74)	80 (16.99)	23 (7.64)*^,^^†^	0.016
Diabetes [*n* (%)]	52 (4.19)	6 (1.28)	4 (0.85)	42 (13.95)***^,^^†††^	<0.001
Hypertension [n (%)]	244 (19.68)	54 (11.54)	73 (15.50)	117 (38.87)***^,^^†††^	<0.001
Family history of CVD [*n* (%)]	67 (5.40)	26 (5.56)	23 (4.88)	18 (5.98)	0.868
Use antihypertensive agents [*n* (%)]	20 (1.61)	5 (1.07)	6 (1.27)	9 (2.99)	0.052
Use lipid-lowering agents [*n* (%)]	10 (0.81)	2 (0.43)	4 (0.85)	4 (1.33)	0.171
Use antidiabetic agents [*n* (%)]	18 (1.45)	5 (1.07)	5 (1.06)	8 (2.66)	0.097
Laboratory data					
Homocysteine (μmol/L)	11.97 (9.80, 14.83)	11.67 (9.62, 14.33)	11.97 (9.83, 14.89)	12.49 (9.91, 15.36)*	0.019
HbA1c (%)	5.52 ± 0.68	5.33 ± 0.55	5.48 ± 0.63**	5.87 ± 0.80***^,^^†††^	<0.001
FBG (mmol/L)	5.36 ± 1.01	5.10 ± 0.70	5.45 ± 0.99***	5.63 ± 1.29***^,^^†^	<0.001
Uric acid (μmol/L)	387.35 ± 97.58	399.07 ± 101.17	386.17 ± 97.86	370.97 ± 88.90***^,^^†^	<0.001
TC (mmol/L)	5.35 ± 0.98	5.29 ± 0.98	5.33 ± 0.95	5.48 ± 1.04**^,^^†^	0.009
TG (mmol/L)	1.29 (0.92, 1.93)	1.27 (0.92, 1.96)	1.28 (0.90, 1.91)	1.29 (0.98, 1.93)	0.626
HDL-C (mmol/L)	1.23 ± 0.30	1.21 ± 0.28	1.22 ± 0.31	1.25 ± 0.29	0.069
LDL-C (mmol/L)	3.36 ± 0.87	3.36 ± 0.85	3.35 ± 0.86	3.35 ± 0.90	0.917
Apo-A (g/L)	1.47 ± 0.23	1.43 ± 0.22	1.45 ± 0.23	1.53 ± 0.25***^,^^†††^	<0.001
Apo-B (g/L)	1.01 ± 0.26	0.99 ± 0.26	1.00 ± 0.25	1.05 ± 0.27**^,^^†^	0.005
Lp(a) (mg/L)	162.00 (91.00, 284.50)	150.00 (80.00, 267.00)	159.00 (98.25, 282.75)	183.00 (101.00, 314.00)*	0.042
eGFR (ml/min·per 1.73 m^2^)	103.94 ± 13.72	113.02 ± 11.57	102.50 ± 10.93***	92.04 ± 10.34***^,^^†††^	<0.001
Cystatin C (mg/L)	0.92 ± 0.18	0.86 ± 0.14	0.89 ± 0.15*	1.03 ± 0.21***^,^^†††^	<0.001

Variables are shown as means ± SD, medians (interquartile range), or absolute numbers and percentages. SBP, systolic blood pressure; DBP, diastolic blood pressure; BMI, body mass index; CVD, cardiovascular diseases; HbA1c, glycated hemoglobin A1c; FBG, fasting blood glucose; TC, total cholesterol; LDL-C, low-density lipoprotein cholesterol; HDL-C, high-density lipoprotein cholesterol; TG, triglyceride; Apo-A, apolipoprotein A-1; Apo-B, apolipoprotein B100; Lipoprotein (a), Lp(a); eGFR, estimated glomerular filtration rate.

* *P* < 0.05.

** *P* < 0.01.

*** *P* < 0.001 vs. 20–39-year-old group.

^†^ *P* < 0.05.

^††^ *P* < 0.01.

^†††^ *P* < 0.001 vs. 40–59-year-old group.

### 3.2 Skin AGEs and Framingham ASCVD risk score

As shown in Table 2, an increasing trend of skin AGEs was observed from Group Ⅰ, Group Ⅱ to Group Ⅲ (66.16 ± 7.06 AU vs. 73.07 ± 10.42 AU vs. 81.84 ± 12.95 AU, respectively, *P* for trend <0.001). The Framingham ASCVD risk score also elevated gradually across age groups [2.80(1.89, 4.12)% vs. 6.41(3.76, 10.67)% vs. 16.34(10.84, 25.39)%, respectively, *P* for trend <0.001]. The prevalence of low ASCVD risk displayed a downward trend from young to old (98.72% vs. 71.34% vs. 23.26%, *P* for trend <0.001). Conversely, the prevalence of medium and high ASCVD risk displayed an increasing trend in the sequentially increasing age groups (medium risk, 1.07% vs. 24.20% vs. 37.87%; high risk, 0.21% vs. 4.46% vs. 38.87%; both *P* for trend <0.001).

**Table 2:** Skin AGEs and Framingham ASCVD risk score and related risk classification.

Variables	All subjects (*n* = 1,240)	20–39 age years old (*n* = 468)	40–59 age years old (*n* = 471)	60–79 age years old (*n* = 301)	*P* for trend
AGEs (AU)	72.59 ± 11.71	66.16 ± 7.06	73.07 ± 10.42***	81.84 ± 12.95***^,^^†††^	<0.001
Framingham ASCVD risk score (%)	5.35 (2.81, 11.57)	2.80 (1.89, 4.12)	6.41 (3.76, 10.67)***	16.34 (10.84, 25.39)***^,^^†††^	<0.001
Low ASCVD risk [*n* (%)]	868 (70.00)	462 (98.72)	336 (71.34)*	70 (23.26)***^,^^†††^	<0.001
Medium ASCVD risk [*n* (%)]	233 (18.79)	5 (1.07)	114 (24.20)***	114 (37.87***^,^^†^	<0.001
High ASCVD risk [*n* (%)]	139 (11.21)	1 (0.21)	21 (4.46)***	117 (38.87)***^,^^†††^	<0.001

Variables are shown as means ± SD, medians (interquartile range), or absolute numbers and percentages. AGEs, advanced glycation end-products; ASCVD, atherosclerotic cardiovascular disease.

* *P* < 0.05.

** *P* < 0.01.

*** *P* < 0.001 vs. 20–39-year-old group.

^†^ *P* < 0.05.

^††^ *P* < 0.01.

^†††^ *P* < 0.001 vs. 40–59-year-old group.

### 3.3 Relationship of skin AGEs and Framingham ASCVD risk score

**Figure 2:**
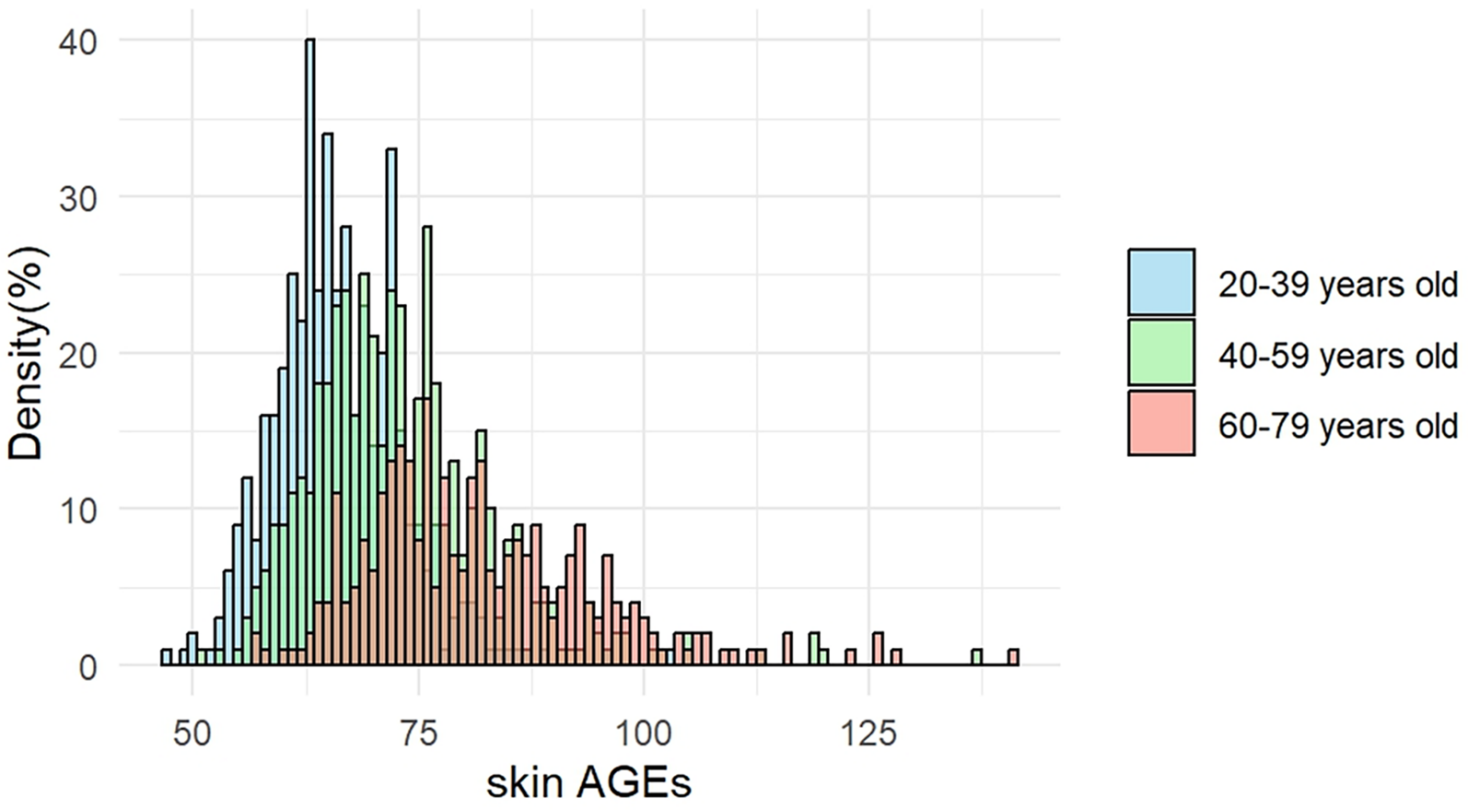
Age-stratified distribution of skin AGEs. AGEs, advanced glycation end-products.

**Figure 3:**
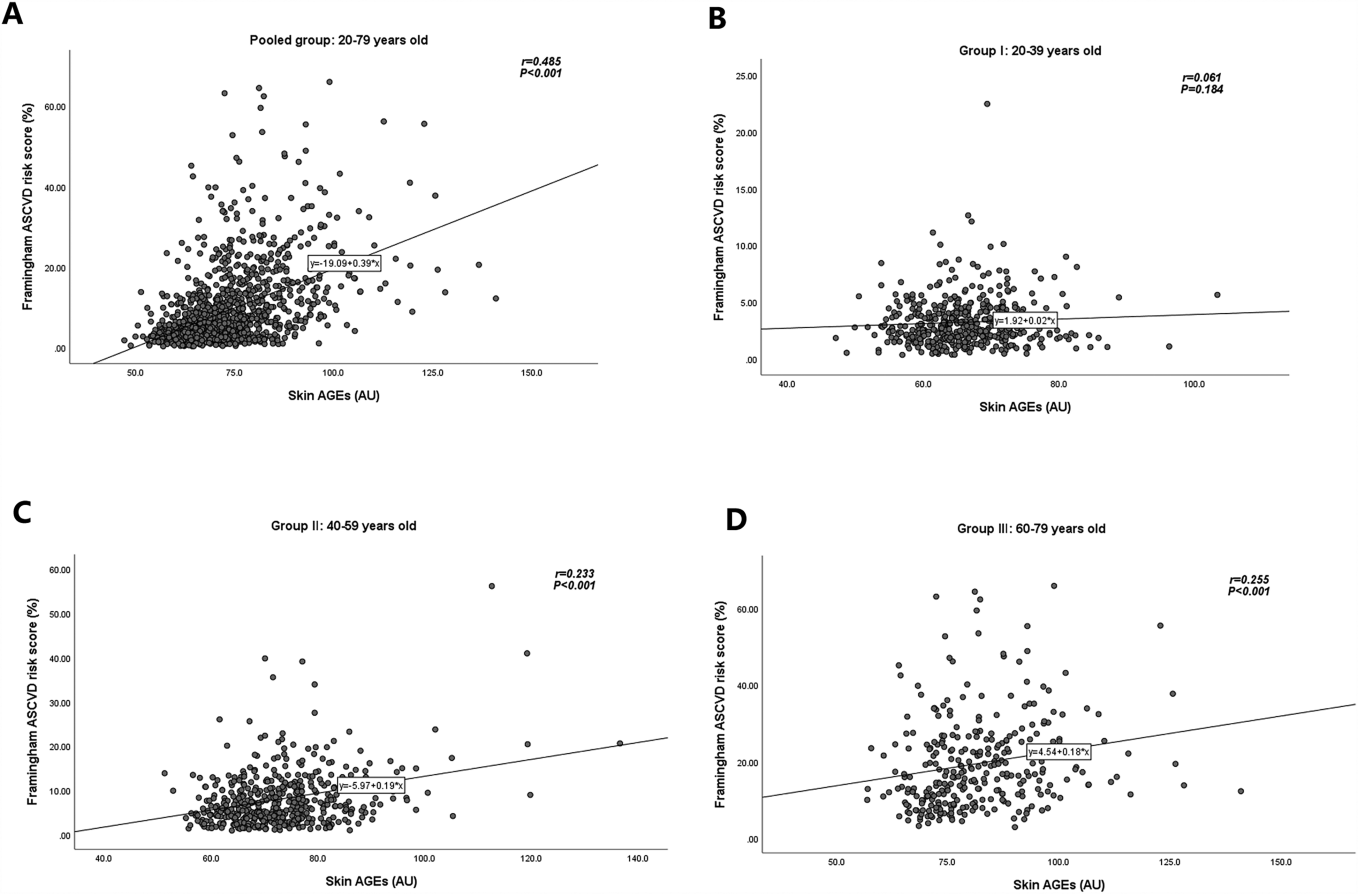
Relationships of skin AGEs and Framingham ASCVD risk score in different age groups. **(A)** Pooled group: 20–79 years old. **(B)** Group Ⅰ: 20–39 years old. **(C)** Group Ⅱ: 40–59 years old. **(D)** Group Ⅲ: 60–79 years old. AGEs, advanced glycation end-products; ASCVD, atherosclerotic cardiovascular disease.

The distribution of skin AGEs by age group is shown in Figure 2. As shown in Figures 3A–D, skin AGEs were correlated with Framingham ASCVD risk score significantly in the pooled group (*r* = 0.485, *P* < 0.001). In the subgroup analysis, the positive correlations remained in Group Ⅱ and Group Ⅲ (*r* = 0.233 and *r* = 0.255, respectively, *P* < 0.001).

### 3.4 Ordinal logistic regression analysis for skin AGEs and Framingham ASCVD risk categories

Univariate logistic regression analysis revealed that skin AGEs were significantly associated with the presence of ASCVD risk in the pooled group (*P* < 0.001; Table 3). Among the potential ASCVD risk factors, only BMI, Hcy, uric acid, TG, LDL-C, Apo-B, eGFR, and CysC were significantly associated with ASCVD risk, respectively, as shown in Supplementary Table S1. Further adjusting for the above risk factors, skin AGEs were still significantly associated with the presence of ASCVD risk (OR 1.029, 95% CI 1.003–1.056, *P* = 0.018; Table 3 and Supplementary Table S2). Interaction analysis indicated that the interaction terms between skin AGEs and age groups were statistically significant (Supplementary Table S3), suggesting that the relationship between skin AGEs and ASCVD risk may differ across age groups. To distinguish the influence of different age ranges, we further performed subgroup regression analysis stratified by age. In Group Ⅱ and Group Ⅲ, skin AGEs were significantly associated with the ASCVD risk (all *P* < 0.05; Table 3). Similarly, the multivariate-adjusted odds ratios of ASCVD risk in Group Ⅱ and Group Ⅲ were 1.047 (95% CI 1.025–1.069) and 1.022 (95% CI 1.002–1.042), respectively.

**Table 3:** Univariate and multivariate logistic regression analysis for skin AGEs and Framingham ASCVD risk category.

Group	ASCVD risk (%)	
	Odds ratio (95%CI)	*P* value
All subjects (*n* = 1,240)		
Univariate	1.049 (1.029, 1.070)	<0.001
Multivariate	1.029 (1.003, 1.056)	0.018
20–39 age years old (*n* = 468)		
Univariate	1.006 (0.899, 1.126)	0.913
Multivariate	0.948 (0.724, 1.240)	0.696
40–59 age years old (*n* = 471)		
Univariate	1.052 (1.032, 1.073)	<0.001
Multivariate	1.047 (1.025, 1.069)	<0.001
60–79 age years old (*n* = 301)		
Univariate	1.028 (1.011, 1.045)	0.001
Multivariate	1.022 (1.002, 1.042)	0.031

AGEs, advanced glycation end-products; ASCVD, atherosclerotic cardiovascular disease.

The multivariable-adjusted model was adjusted for the risk factors unrelated to the formula of Framingham ASCVD risk score including age group, body mass index, homocysteine, uric acid, triglyceride, low-density lipoprotein cholesterol, apolipoprotein B100, estimated glomerular filtration rate, and cystatin C.

Multicollinearity analysis indicated no severe multicollinearity that would distort the model estimates (Supplementary Table S4).

Furthermore, RCS was employed to assess non-linear associations between skin AGEs and ASCVD risk in a multivariable-adjusted model. The results indicated a significant overall association (*P*_overall_ < 0.001) but no evidence of non-linearity (P_non-linear_ = 0.735), suggesting that the relationship between skin AGEs and ASCVD risk was linear (Supplementary Figure S1).

### 3.5 ROC curve analysis for predictive values of skin AGEs in detecting ASCVD risk

**Figure 4:**
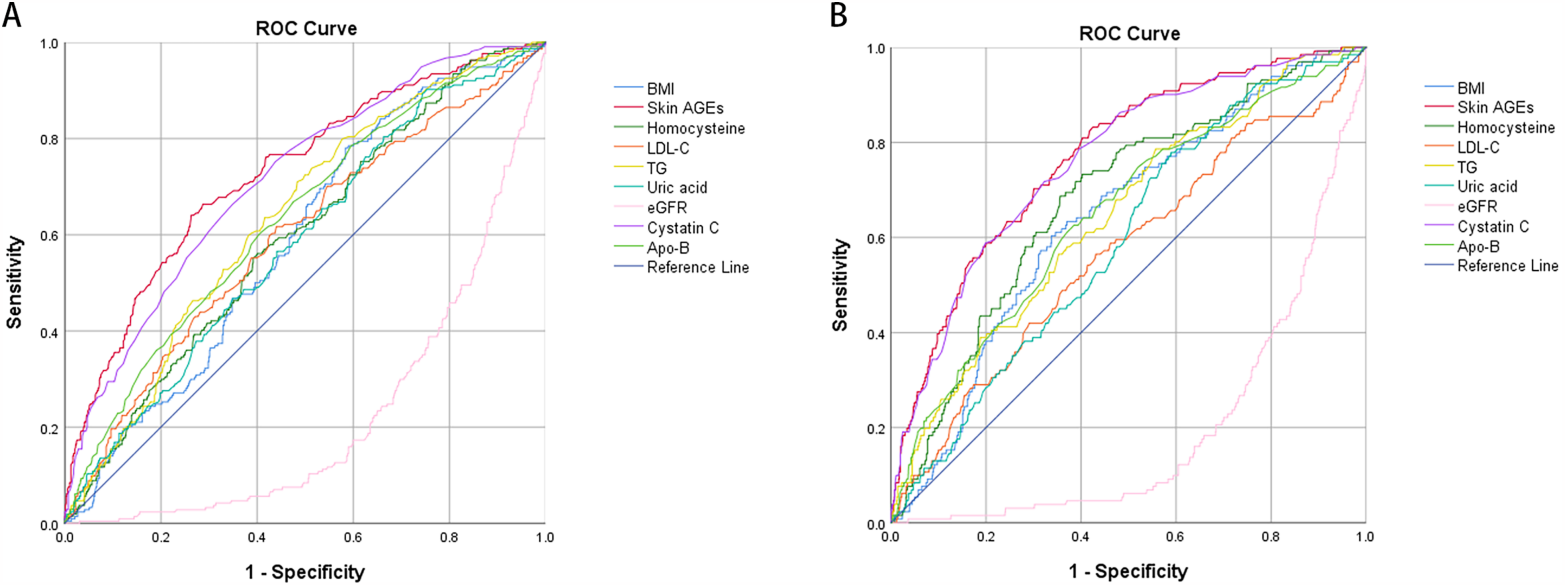
Comparison of receiver-operating characteristic (ROC) curve analysis of different parameters in detecting the ASCVD risk in the pooled group. **(A)** The analysis was for medium ASCVD risk. **(B)** The analysis was for high ASCVD risk. ASCVD, atherosclerotic cardiovascular disease; BMI, body mass index; AGEs, advanced glycation end-products; LDL-C, low-density lipoprotein cholesterol; TG, triglyceride; Apo-B, apolipoprotein B100; eGFR, estimated glomerular filtration rate.

**Figure 5:**
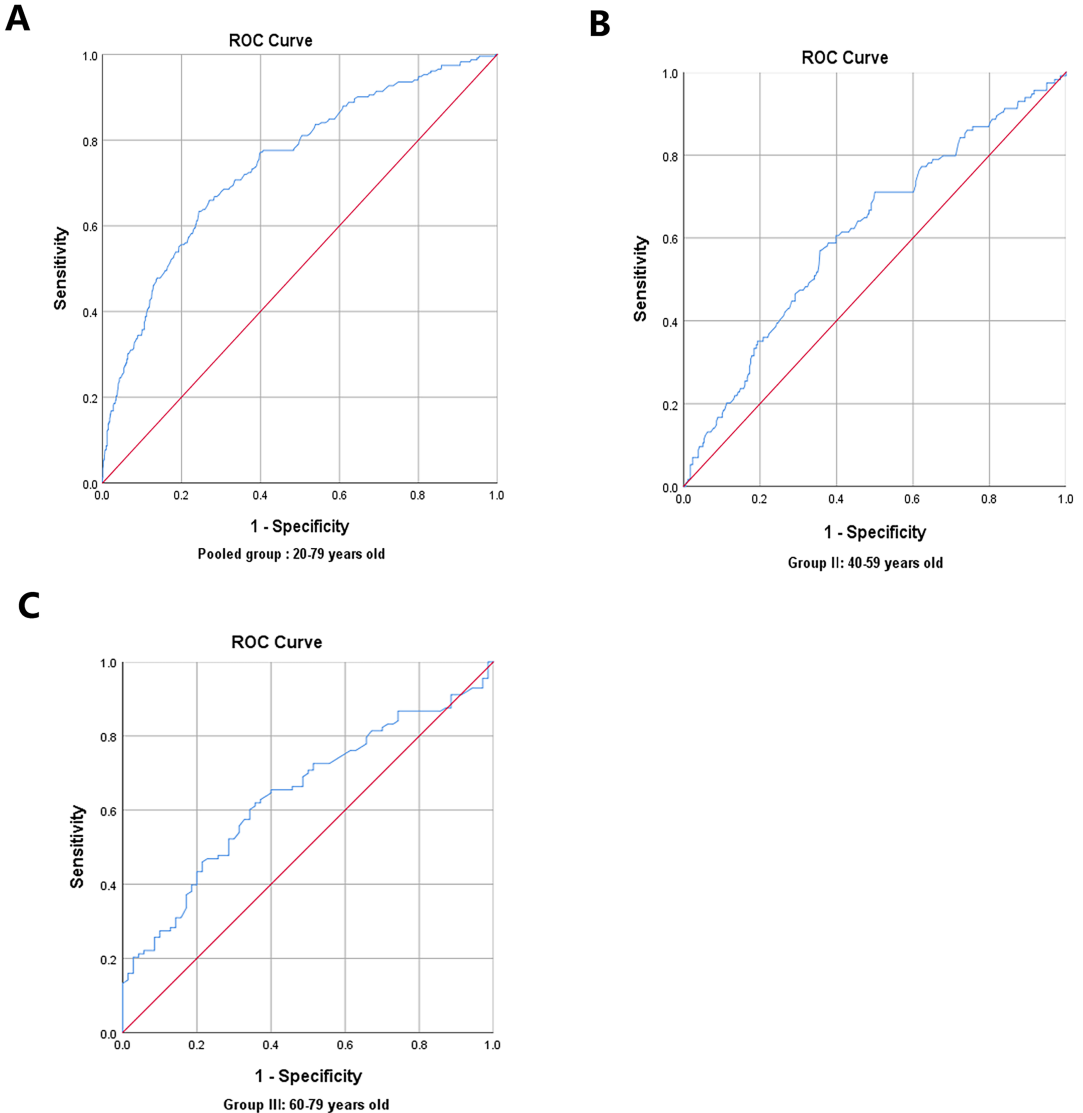
Receiver-operating characteristic (ROC) curve analysis for predictive values of skin AGEs in detecting medium ASCVD risk in different age groups. **(A)** Pooled group: 20–79 years old. **(B)** Group Ⅱ: 40–59 years old. **(C)** Group Ⅲ: 60–79 years old. AGEs, advanced glycation end-products; ASCVD, atherosclerotic cardiovascular disease.

**Figure 6:**
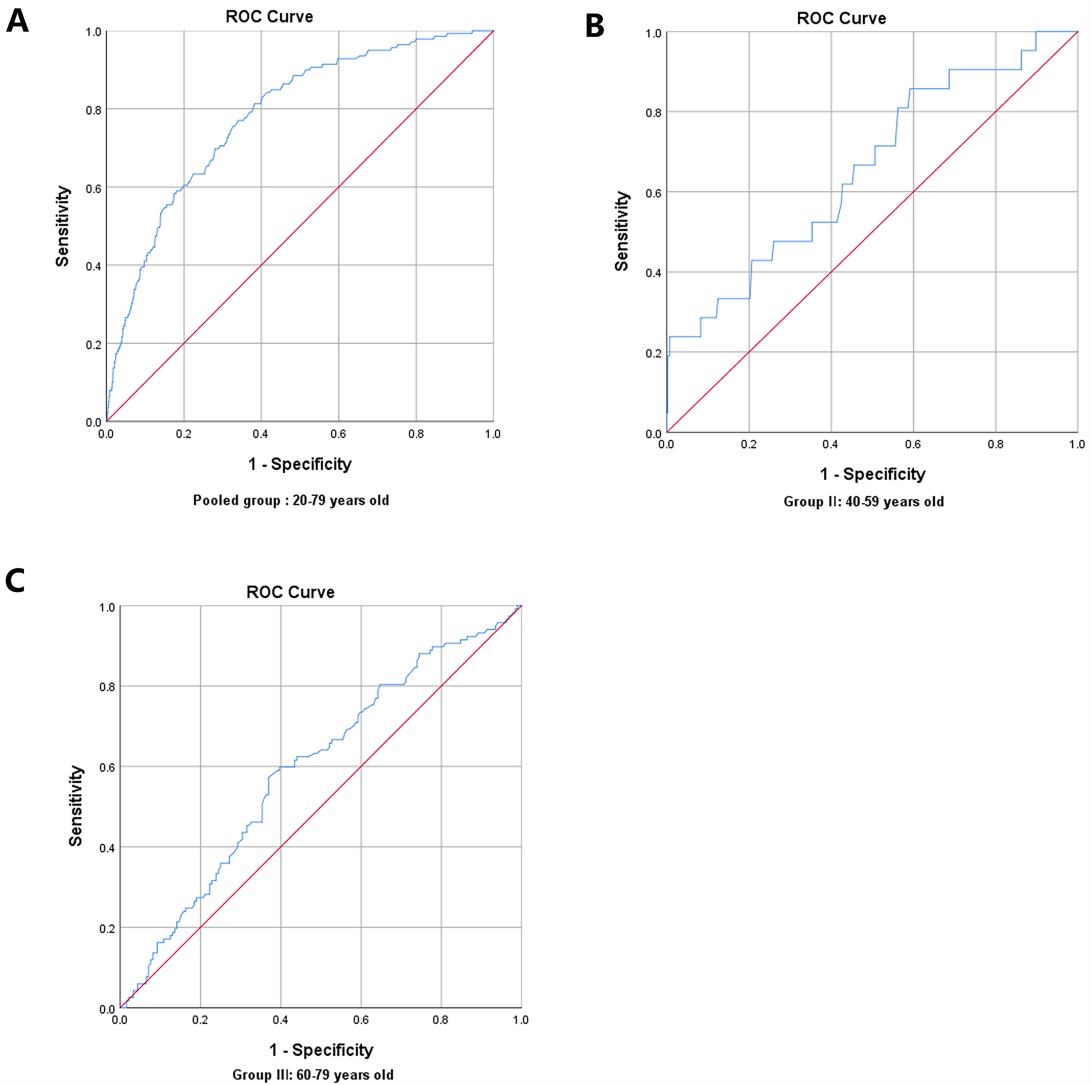
Receiver-operating characteristic (ROC) curve analysis for predictive values of skin AGEs in detecting high ASCVD risk in different age groups. **(A)** Pooled group: 20–79 years old. **(B)** Group Ⅱ: 40–59 years old. **(C)** Group Ⅲ: 60–79 years old. AGEs, advanced glycation end-products; ASCVD, atherosclerotic cardiovascular disease.

In the pairwise comparisons of the ROC curves among the abovementioned potential ASCVD risk factors, skin AGEs were superior to any other risk factors, all *P* < 0.05, as seen in Figures 4A,B. Furthermore, the area under the ROC curve for skin AGEs in detecting the presence of medium or high ASCVD risk in the pooled group, Group Ⅱ, and Group Ⅲ, was statistically significant, all *P* < 0.05, as shown in Figures 5A–C, Figures 6A–C, and Table 4, respectively. Based on the ROC curve, the optimal cutoff for skin AGEs in detecting the presence of medium ASCVD risk in the pooled group, Group Ⅱ, and Group Ⅲ was 73.60, 73.60, and 77.25 AU, respectively. The optimal cutoff for skin AGEs in detecting the presence of high ASCVD risk in the pooled group, Group Ⅱ, and Group Ⅲ was 72.35, 69.25, and 81.35 AU, respectively.

**Table 4:** The ROC results of skin AGEs predicting Framingham ASCVD risk.

Group	Cutoff point	Sensitivity (%)	Speciﬁcity (%)	AUC	*P* value
All subjects					
Medium ASCVD risk (%)	73.60AU	63.36%	75.58%	0.744 (0.707–0.780)	<0.001
High ASCVD risk (%)	72.35AU	81.29%	61.76%	0.783 (0.744–0.821)	<0.001
40–59 age years old					
Medium ASCVD risk (%)	73.60AU	57.02%	64.29%	0.613 (0.553–0.673)	<0.001
High ASCVD risk (%)	69.25AU	61.95%	64.29%	0.643 (0.563–0.723)	0.001
60–79 age years old					
Medium ASCVD risk (%)	77.25AU	85.71%	40.89%	0.657 (0.535–0.779)	0.015
High ASCVD risk (%)	81.35AU	57.26%	63.04%	0.594 (0.529–0.659)	0.006

ROC, receiver-operating characteristic; AGEs, advanced glycation end-products; ASCVD, atherosclerotic cardiovascular disease; AUC, area under the curve.

## 4 Discussion

The present study revealed a discernible trend of increasing skin AGE accumulation across different age groups, with significant associations between skin AGEs and ASCVD risk, irrespective of proven cardiovascular risk factors. These findings underscore the potential of skin AGEs as a novel indicator for ASCVD risk assessment, especially in individuals aged 40–59 and 60–79.

AGE accumulation is a well-documented phenomenon associated with aging, primarily due to increased lifelong exposure to glycation (2). The progressive increase in AGEs might reflect cumulative metabolic and environmental stress, thereby serving as a risk factor for cardiovascular aging (7). With advancing research, an increasing number of studies have started to explore the connection between AGEs and cardiovascular diseases, considering AGEs as a potential indicator for assessing ASCVD (16–18).

Previous studies have shown mixed results regarding the predictive value of AGEs for cardiovascular events. While some studies were accordant with our findings, indicating a strong correlation between AGEs and cardiovascular risks (19), others suggest a more nuanced relationship, possibly influenced by factors such as diabetes status, renal function, and lifestyle factors (20). Recently, a longitudinal study, using the Framingham risk score as the surrogate indicator of cardiovascular disease risk similar to our study, found that AGEs were independently associated with the Framingham ASCVD risk score in the Mexican population (14). However, unlike our study where AGEs were measured by SAF, they adopted serum AGE level as a potential risk factor to explore the association between AGEs and ASCVD. Serum AGEs are mainly detected in the free state or bound to short-half-life proteins, which reflect their levels over the short term (days or weeks) (21). However, serum AGEs can be influenced by various factors, including diet, metabolic state, kidney function, and the individual's ability to clear AGEs (22), and may not directly reflect the accumulation of AGEs in tissues or organs. In contrast, skin AGEs predominantly represent stable collagen cross-linking products that accumulate progressively over months to years (22). This compartmentalization reflects long-term cumulative AGE exposure and demonstrates superior biological persistence compared to circulatory biomarkers (22). Prior investigations have confirmed that there is no significant correlation between skin AGEs and serum AGEs (23, 24). In addition, several clinical studies found that skin AGEs assessed by SAF were more advantageous than serum AGEs in the general population (23, 25). Measuring AGEs in tissues can provide direct evidence of AGEs accumulation in specific organs or tissues, but this method is generally invasive and not suitable for routine or repeated measurements. Differently, SAF is a non-invasive measurement method used to assess the level of AGEs accumulated in the skin. Although SAF may not fully correspond to the levels of AGEs in tissues or blood, it can provide a convenient way to estimate the total burden of AGEs in the body. Since the protein turnover rate in the skin is slow, the accumulation of AGEs in the skin may reflect a long-term metabolic effect (9, 22). So it usually is used as a surrogate indicator of circulating or tissue AGEs to study the pathogenic effect of AGEs on diabetes and its complications or cardiovascular diseases (12, 26). Unlike previous studies about AGEs and ASCVD, we performed a subgroup analysis stratified by 20-year age intervals to find the exact association of AGEs and ASCVD across different age groups. Just like SAF has been used to definite predictions of diabetes models and related diagnostic thresholds in clinical practice, SAF may similarly have an important diagnostic value in ASCVD. We expected to determine the predictive effect on ASCVD risk and provide the diagnostic thresholds according to different age intervals for clinical workers. That was exactly what we wanted to do.

The Framingham ASCVD risk score is an easy and convenient tool to fast discriminate ASCVD stratification in large general people or participants for physical examination. To date, studies incorporating skin AGE measurement into the Framingham ASCVD risk assessment model are not common. The Framingham Heart Study primarily focuses on traditional cardiovascular risk factors, such as age, gender, SBP, TC, HDL-C, smoking, hypertension, and diabetes (11). Except for mentioned above risk factors, BMI, TG, LDL-C, Apo-B, Hcy, uric acid, eGFR, and CysC were also the proven risk factors for ASCVD (27–31) and had statistically significant correlations with Framingham ASCVD risk in the present study, shown in Supplementary Table S1. Therefore, they were incorporated into regression models to clarify the contribution role of AGEs to ASCVD risk. Adjusting for the effects of BMI, TG, LDL-C, Apo-B, Hcy, uric acid, eGFR, and CysC, the ASCVD risk was elevated by 2.9%. Although the ASCVD risk elevated slightly, the results still suggested that underlying damage to the cardiovascular system was not fully captured by common risk factors, and skin AGEs could provide additional predictive value beyond the established cardiovascular risk markers.

According to the multivariate regression analysis stratified by age, the ASCVD risk elevated 4.7% and 2.2% per 1 AU increase of SAF for 40–59-year-old and 60–79-year-old people, respectively. Nevertheless, the odds ratios of skin AGEs predicting ASCVD risk in the 20–39 age group were not statistically significant. In younger individuals (aged 20–39), the body's defense mechanisms against glycation and oxidative stress may still effectively counteract the detrimental effects of AGE accumulation. This resilience might explain the lack of a significant association between skin AGEs and ASCVD risk in this age group. Additionally, the medium or high risk of ASCVD in younger individuals is fairly lower in our study, which could further obscure the impact of AGEs on cardiovascular risk. In contrast, the significant associations observed in the 40–59 and 60–79 age groups might reflect a tipping point where the cumulative burden of AGEs begins to overwhelm the body's defensive mechanisms, leading to noticeable effects on vascular function and structure. This period coincides with the onset of various age-related physiological changes, including reduced elasticity of blood vessels, diminished endothelial function, and increased inflammatory responses (5, 32), all of which are known to be influenced by AGEs. The significant associations between skin AGEs and ASCVD risk in middle-aged and older populations suggest that AGEs could serve as a marker of accelerated vascular aging and an independent risk factor for ASCVD. Meanwhile, the different associations across different age groups also warn clinicians to pay more attention to preventing ASCVD risk according to age stratification in preventive care.

The potential mechanisms of skin AGEs and ASCVD risk may be as follows. First, AGEs contribute to oxidative stress and inflammatory responses. The interaction between AGEs and their receptor RAGE on cells triggers the activation of NF-kB, leading to the production of pro-inflammatory cytokines and vascular inflammation (32). Second, AGEs can impair endothelial function by reducing the bioavailability of nitric oxide (NO) and enhancing the expression of endothelial adhesion molecules, promoting endothelial cell activation and the recruitment of inflammatory cells, thereby contributing to the development of atherosclerotic lesions (5). Third, the accumulation of AGEs in the vascular wall can induce cross-linking of collagen and elastin fibers, leading to increased arterial stiffness, which is associated with an elevated risk of cardiovascular events (33). Fourth, AGE modification of LDL-C can aggravate atherosclerosis, promoting the formation of foam cells and the development of atherosclerotic plaques. AGEs also impair HDL-C function, which could further exacerbate cardiovascular risk (34). All the above mechanisms constitute the theoretical basis for skin AGEs to independently predict ASCVD risk.

The ROC analysis indicating that skin AGEs could predict medium and high ASCVD risks supported the utility of non-invasive measurement of AGEs as a potential screening tool in clinical practice. SAF may offer a promising avenue for early intervention strategies aimed at reducing ASCVD risk, particularly in populations where traditional risk factors may not fully account for the observed risk. According to the present study, we command 73.60 and 69.25 AU were the optimal cutoff values for skin AGEs in detecting the presence of medium or high ASCVD risk, respectively, for 40–59 years old people, and 77.25 and 81.35 AU were the optimal cutoff value for skin AGEs in detecting the presence of medium or high ASCVD risk, respectively, for 60–79 years old people. Based on these findings, we recommend that clinicians include skin AGE assessment as part of routine checkups, particularly for middle-aged and older individuals. This approach can help identify high-risk patients and provide a foundation for early intervention. Furthermore, these findings highlight skin AGEs’ dual role as both a biomarker and a prevention target for community cardiovascular health. The implementation of age-stratified risk thresholds in ASCVD screening can optimize resource utilization, making the screening process more cost-effective. As a result, it can effectively alleviate the medical burden on the healthcare system and enhance disease early warning and health awareness among the general public (35). The measurement of skin AGEs as a non-invasive and convenient diagnostic tool can offer clinicians additional information regarding patients’ cardiovascular health. By considering skin AGEs alongside other established cardiovascular risk factors, healthcare providers can develop more personalized prevention and treatment strategies. For patients with high AGE levels, clinicians could recommend specific lifestyle modifications, including dietary adjustments, regular exercise, and enhanced blood sugar management. These measures could not only help reduce AGE levels but also improve overall cardiovascular health, thereby decreasing the incidence of ASCVD events.

## 5 Strengths

This study provides novel insights into the age-stratified role of AGEs in ASCVD through several key methodological and analytical strengths. First, by segmenting participants into three distinct age cohorts (20–39, 40–59, 60–79 years), we revealed different associations between AGEs and ASCVD stratified by age, with the associations being more amplified in midlife and older populations. Second, our models comprehensively adjusted for both conventional risk factors such as lipid profile and emerging ASCVD risk factors (homocysteine and cystatin C), confirming the independence of AGE effects.

## 6 Limitations

It should be noted that this study has some limitations. First, the possible causal relationship between skin AGEs and ASCVD could not be clarified due to the cross-sectional study design. Second, this study was a single-center study. The findings would need external validation in independent cohorts to confirm their applicability and reliability across different populations and settings. Future research should focus on elucidating the mechanisms through which AGEs contribute to ASCVD risk and exploring potential interventions to reduce AGEs or their impact on cardiovascular health.

## 7 Conclusion

In summary, the marked associations between skin AGEs and ASCVD risk in individuals aged 40–59 and 60–79 highlight the importance of considering AGEs as a component of ASCVD risk assessment in middle-aged and older adults. As a non-invasive and convenient assessment, SAF enables healthcare practitioners—particularly community healthcare workers—to stratify and manage individuals with elevated ASCVD risk. Incorporating SAF measurement into routine ASCVD risk screening could offer a novel approach to identifying individuals at medium or high risk, particularly in the crucial age above 40, and lead to a more dynamic and precise risk assessment model for age-related ASCVD risks. Extension of this research to multi-ethnic cohorts will validate the external validity of our findings and directly inform population-specific prevention strategies for high-risk subgroups.

## Data Availability

The raw data supporting the conclusions of this article will be made available by the authors, without undue reservation.
