# Supplementary material for: The associations between skin advanced glycation end-products and Framingham cardiovascular risk in different age groups
Source: Front Cardiovasc Med. 2025 Apr 8;12:1491643. doi: 10.3389/fcvm.2025.1491643 (PMC12011794; doi:10.3389/fcvm.2025.1491643)
Supplement: Supplementary file 1 [file Table1.docx]

**Table S1. Univariate logistic regression analysis for clinical variables with Framingham ASCVD risk in all subjects, with data expressed as odds ratio (95%CI)**

|  | ASCVD risk (%) |
| --- | --- |
| Age (years) | 1.158 (1.136 to 1.180)^***^ |
| Sex [F/M (%F)] | 1.579(1.156 to 2.155)^**^ |
| SBP (mmHg) | 1.071(1.059 to 1.084)^***^ |
| DBP (mmHg) | 1.058(1.042 to 1.075)^***^ |
| BMI (kg/m2) | 1.060(1.017 to 1.105)^**^ |
| Smoking [n (%)] | 1.086(0.684 to 1.724) |
| Alcohol drink [n (%)] | 0.916(0.604 to 1.391) |
| Diabetes [n (%)] | 3.810(1.323 to 10.973)^*^ |
| Hypertension [n (%)] | 6.121(4.297 to 8.720)^***^ |
| Family history of CVD [n (%)] | 1.548(0.864 to 2.773) |
| Use anti-hypertension agents [n(%)] | 0.531(0.180 to 1.570) |
| Use lipid-lowering agents [n(%)] | 0.355(0.079 to 1.597) |
| Use anti-diabetic agents [n(%)] | 0.569(0.128 to 2.541) |
| Homocysteine (μmol/L) | 1.030(1.009 to 1.050)^**^ |
| HbA1c (%) | 2.757(2.042 to 3.721)^***^ |
| FBG (mmol/L) | 1.795(1.469 to 2.193)^***^ |
| Uric acid (μmol/L) | 1.003(1.001 to 1.004)^**^ |
| TC (mmol/L) | 1.439(1.237 to 1.675)^***^ |
| TG (mmol/L) | 1.191(1.072 to 1.323)^**^ |
| HDL-C (mmol/L) | 0.323(0.189 to 0.553)^***^ |
| LDL-C (mmol/L) | 1.401(1.183 to 1.660)^***^ |
| Apo-A (g/L) | 0.597(0.309 to 1.153) |
| Apo-B (g/L) | 6.017(3.311 to 10.932)^***^ |
| Lp(a) (mg/L) | 1.000(1.000 to 1.001) |
| eGFR (mL/min·per 1.73 m2 ) | 0.921(0.908 to 0.933)^***^ |
| Cystatin C (mg/L) | 128.954(44.837 to 370.881)^***^ |

ASCVD, atherosclerotic cardiovascular disease; SBP, systolic blood pressure; DBP, diastolic blood pressure; BMI, body mass index; CVD, cardiovascular diseases; HbA1c, glycated hemoglobin A1c; FBG, fasting blood glucose; TC, total cholesterol; LDL-C, low-density lipoprotein cholesterol; HDL-C, high-density lipoprotein cholesterol; TG, triglyceride; Apo-A, apolipoprotein A-1; Apo-B, apolipoprotein B100; Lp(a), Lipoprotein (a); eGFR, estimated glomerular filtration rate. **P*<0.05, ***P*<0.01, ****P*<0.001.
