# Supplementary material for: The associations between skin advanced glycation end-products and Framingham cardiovascular risk in different age groups
Source: Front Cardiovasc Med. 2025 Apr 8;12:1491643. doi: 10.3389/fcvm.2025.1491643 (PMC12011794; doi:10.3389/fcvm.2025.1491643)
Supplement: Supplementary file 2 [file Table2.docx]

**Table S2. Multivariate logistic regression analysis for skin AGEs and Framingham ASCVD risk category in all subjects**

| Variable | Odds Ratio (95%CI) | P value |
| --- | --- | --- |
| skin AGEs (AU) | 1.029 (1.003, 1.056) | 0.018 |
| age group Ⅱ | 13.475 (3.587, 89.758) | <0.001 |
| age group Ⅲ | 89.141 (20.064, 657.751) | <0.001 |
| Homocysteine (μmol/L) | 1.013 (0.969, 1.066) | 0.609 |
| BMI (kg/m²) | 1.108 (1.014, 1.211) | 0.013 |
| Uric acid (μmol/L) | 1.004 (1.001, 1.007) | 0.011 |
| TG (mmol/L) | 1.392 (1.103, 1.794) | 0.007 |
| LDL-C (mmol/L) | 1.573 (1.013, 2.468) | 0.043 |
| Apo-B (g/L) | 2.038 (0.409, 10.114) | 0.381 |
| eGFR (mL/min·per 1.73 m²) | 0.988 (0.961, 1.016) | 0.237 |
| Cystatin C (mg/L) | 11.203 (1.607, 79.057) | 0.004 |
