# Supplementary material for: The associations between skin advanced glycation end-products and Framingham cardiovascular risk in different age groups
Source: Front Cardiovasc Med. 2025 Apr 8;12:1491643. doi: 10.3389/fcvm.2025.1491643 (PMC12011794; doi:10.3389/fcvm.2025.1491643)
Supplement: Supplementary file 3 [file Table3.docx]

**Table S3—Interaction analysis of skin AGEs and age groups for ASCVD risk**

| Term | Odds Ratio (95%CI) | *P* value |
| --- | --- | --- |
| Skin AGEs (AU) | 1.006 (0.991, 1.022) | 0.421 |
| Age group Ⅱ | 1.278 (0.443, 3.685) | 0.650 |
| Age group Ⅲ | 40.884 (14.479, 115.439) | <0.001 |
| Skin AGEs: age group Ⅱ | 1.043 (1.024, 1.062) | <0.001 |
| Skin AGEs: age group Ⅲ | 1.023 (1.006, 1.041) | 0.009 |
