# Supplementary material for: The associations between skin advanced glycation end-products and Framingham cardiovascular risk in different age groups
Source: Front Cardiovasc Med. 2025 Apr 8;12:1491643. doi: 10.3389/fcvm.2025.1491643 (PMC12011794; doi:10.3389/fcvm.2025.1491643)
Supplement: Supplementary file 4 [file Table4.docx]

**Table S4. Multicollinearity analysis**

| Variable | VIF | VIF^(1/(2*Df))^ |
| --- | --- | --- |
| skin AGEs (AU) | 1.18 | 1.09 |
| age group | 1.68 | 1.14 |
| Homocysteine (μmol/L) | 1.11 | 1.05 |
| BMI (kg/m²) | 1.26 | 1.12 |
| Uric acid (μmol/L) | 1.45 | 1.2 |
| TG (mmol/L) | 1.64 | 1.28 |
| LDL-C (mmol/L) | 3.05 | 1.75 |
| Apo-B (g/L) | 3.01 | 1.74 |
| eGFR (mL/min·per 1.73 m²) | 1.48 | 1.21 |
| Cystatin C (mg/L) | 1.38 | 1.17 |

A VIF value exceeding 5 or GVIF ^(1/(2*Df))^ value exceeding 2 indicates potential multicollinearity.
