# Supplementary material for: The associations between skin advanced glycation end-products and Framingham cardiovascular risk in different age groups
Source: Front Cardiovasc Med. 2025 Apr 8;12:1491643. doi: 10.3389/fcvm.2025.1491643 (PMC12011794; doi:10.3389/fcvm.2025.1491643)
Supplement: Supplementary file 5 [file Image1.pdf]

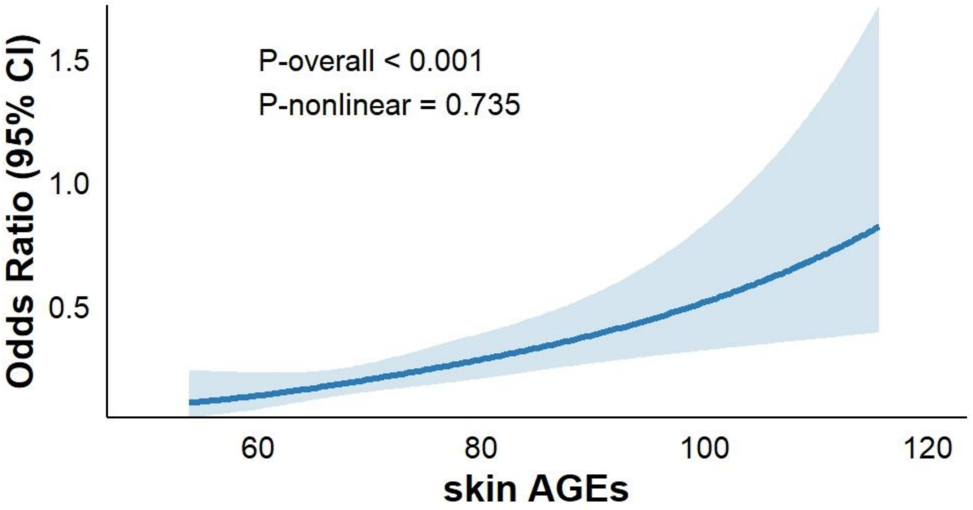

Supplementary Figure S1. Restricted cubic splines (RCS) analysis between skin AGEs and ASCVD risk in multivariable-adjusted model.

Multivariable-adjusted model was adjusted for the risk factors unrelated with the formula of Framingham ASCVD risk score including age group, body mass index, homocysteine, uric acid, triglyceride, low-density lipoprotein cholesterol, apolipoprotein B100, estimated glomerular filtration rate and Cystatin C.
